# Properties and Interfacial Bonding Enhancement of Oil Palm Bio-Ash Nanoparticles Biocomposites

**DOI:** 10.3390/polym13101615

**Published:** 2021-05-17

**Authors:** C. K. Abdullah, I. Ismail, M. R. Nurul Fazita, N. G. Olaiya, H. Nasution, A. A. Oyekanmi, Arif Nuryawan, Abdul Khalil H. P. S.

**Affiliations:** 1 School of Industrial Technology, Universiti Sains Malaysia, Penang 11800, Malaysia; abdulkan2000@yahoo.com; 2 Physics Department, Mathematics and Natural Sciences Faculty, Universitas Syiah Kuala, Banda Aceh 23111, Indonesia; ismailab@unsyiah.ac.id; 3 Department of Industrial and Production Engineering, Federal University of Technology, Akure PMB 704, Nigeria; ngolaiya@futa.edu.ng; 4 Department of Chemical Engineering, Faculty of Engineering, Universitas Sumatera Utara, Padang Bulan, Medan 20155, Indonesia; halimatuddahliana@usu.ac.id; 5 Department of Forest Products Technology, Faculty of Forestry, Universitas Sumatera Utara, Medan 20155, Indonesia; Arif5@usu.ac.id

**Keywords:** oil palm bio-ash, agriculture waste, material characterizations, nanoparticle, nano biocomposite

## Abstract

The effect of incorporating different loadings of oil palm bio-ash nanoparticles from agriculture waste on the properties of phenol-formaldehyde resin was investigated in this study. The bio-ash filler was used to enhance the performance of phenol-formaldehyde nanocomposites. Phenol-formaldehyde resin filled with oil palm bio-ash nanoparticles was prepared via the in-situ polymerization process to produce nanocomposites. The transmission electron microscope and particle size analyzer result revealed that oil palm bio-ash nanoparticles had a spherical geometry of 90 nm. Furthermore, X-ray diffraction results confirmed the formation of crystalline structure in oil palm bio-ash nanoparticles and phenol-formaldehyde nanocomposites. The thermogravimetric analysis indicated that the presence of oil palm bio-ash nanoparticles enhanced the thermal stability of the nanocomposites. The presence of oil palm bio-ash nanoparticles with 1% loading in phenol-formaldehyde resin enhanced the internal bonding strength of plywood composites. The scanning electron microscope image revealed that phenol-formaldehyde nanocomposites morphology had better uniform distribution and dispersion with 1% oil palm bio-ash nanoparticle loading than other phenol-formaldehyde nanocomposites produced. The nanocomposite has potential use in the development of particle and panel board for industrial applications.

## 1. Introduction

Oil palm plantations are abundant and available globally. All part of the oil palm is useful for domestic purposes. However, most oil palm plant parts are disposed of as waste biomass because of their abundance. The large production of palm oil by oil extraction in the palm oil mill has abundantly produced secondary waste, negatively impacting health due to environmental pollution [1]. Most of the oil palm wastes from palm oil production are currently burned to generate steam for sterilization. The combustion of mesocarp palm fibre and palm kernel shells generate steam in a palm oil mill, resulting in an excessive amount of oil palm ash or boiler ash as secondary wastes [2]. Moreover, the boiler incineration process has raised environmental concerns due to the release of flue gases containing ash, CO, and NO_2_ into the atmosphere [3]. The oil palm ash with the light greyish colour is the waste often obtained from palm oil extraction and dumped behind the mill as unutilized left-over [4].

Nanoparticles have been used as filers for improved physical and chemical strength, and thermal properties of polymers [5]. Nanoparticles derived from renewable sources have also been used to improve the degradation kinetics of synthetic polymers [6]. OPB nanoparticles have been used in this respect as a filler to reduce the volatilization of the polymer thermal decomposition, which enhances their stability [7]. The oil palm bio-ash (OPB) is a mixture of particles with very complex compositions. Many studies have evaluated the usefulness of oil palm ashes due to the high concentration of various oil palm oil elements. Previous studies have shown the possibility of using OPB as a bio-absorbent material [8], bleaching absorbent [2], partial sand replacement in concrete [4,9], nano-engineered wood-based panel [10], nanoparticles reinforcement materials [11,12], and as an adsorbent material for the removal of toxic element in contaminated water [13,14]. Although various oil palm ash applications have been explored, a significant quantity of OPA is still disposed to landfills, requiring a lot of land area and resulting in a huge pile of solid wastes [8,15]. To minimize the environmental problems caused by oil palm ash production, the potential to integrate this material in polymer mixing materials is deemed as a viable option to prevent the excessive use of the earth’s non-renewable assets.

In thermoset polymers, phenol-formaldehyde (PF) resin is one of the most common formaldehyde-based resins, which provides outstanding adhesion performance, high-temperature resistance, flame resistance, and electric insulation. Thus, phenol-formaldehyde (PF) resins have been and are still being used extensively in the industry [16,17]. Besides, the PF resin is a widely used wood adhesive in the industrial production of exterior-grade plywood panels, oriented strand board panels, and particleboards due to its excellent strength, temperature stability and resistance to moisture [18]. Phenol-formaldehyde condensation products have one of the lowest cost productions, totaling over 500,000 metric tonnes per year [19]. At present, its estimated market value has risen to $10 billion globally, with an annual range between $4.5 billion to $6 billion [20]. The utilization of palm oil industry waste in a power plant such as oil palm kernel shell [21], empty fruit bunch [22,23], and palm oil mill effluent [24] has been reported as added values. Indonesia and Malaysia produce about 90% of the world’s OP waste. An estimated 51.19 million tonnes (Mt) of oil palm waste was generated in Malaysia alone [25].

Despite the advantages of phenol-formaldehyde resin, its major drawbacks prevent its widespread application. Some of the challenges attributed to PF resins are high brittleness and shrinkage. Improvement in the properties and performance of resin composite has been intensively investigated in recent years. It was proposed that the addition of a nanofiller can enhance its functional properties, such as permeability resistance, improved thermal stability, enhanced mechanical properties, and decreased the formaldehyde emission values [26]. Some researchers have introduced nanomaterials into phenolic resins to improve their thermal stability because non-penetrable glass coatings are believed to be formed during thermal degradation. The glass coatings effectively exclude oxygen from the phenolic resins and prevent the material’s combustion [6,27]. The improved thermal stability was ascribed to a better interfacial interaction between nanomaterials and the PF resin matrix [17].

The suitability of oil palm bio-ash incorporated into phenolic adhesives is partly determined by chemical features involved in polymerization reactions. The two most important oil palm ash features are bonding with the phenolic hydroxyl and the main element composition in oil palm ash. Generally, the addition of fillers can enhance PF resin with specific properties. However, the addition of fillers sometimes leads to the degradation of the characteristics of polymers. However, the appearance of nanotechnology opens a new route for improving PF resin’s properties [28]. To improve the limitations of previous works, this study focuses on investigating the role of oil palm bio-ash (OPB) in phenol-formaldehyde (PF) resin. PF nanocomposites’ properties filled OPB nanoparticles at five different loadings (1% to 5%). Thus, the main objective of the present study was to investigate the effects of OPB nanoparticles with the incorporation of different loadings on molecular structure, thermal, crystallinity, and morphological properties of phenol-formaldehyde nanocomposites-filled OPB nanoparticles.

PF resin is noted for low internal bonding strength and thermal stability when used in panelboards [29,30]. The incorporation of nanoparticles from a renewable source was used in this study to enhance these properties. The incorporation of nanoparticles from OPB as a filler in PF resin to form a nanocomposite for improved internal bonding and thermal properties has not been studied. Furthermore, incorporating OPB nanoparticles into the PF resin without adding any additives or miscibility enhancer has not been reported. The properties of nanocomposites with different percentage loadings of nanofiller was studied for improved thermal stability, strength and permeability resistance.

## 2. Materials and Methods

### 2.1. Materials

Oil palm bio-ash (OPB) was obtained from United Oil Palm Mill, Penang, Malaysia. The bio-waste from the hopper section, also known as secondary waste in the palm oil mill, was a suitable candidate due to its particle size, which is more effortless to convert from micron to nano-size materials. The polymeric matrix used was phenol-formaldehyde (PF) resin, a resol type used specifically for wood-based applications. The resin was obtained from Hexion Specialty Chemicals Sdn. Bhd., Penang, Malaysia.

### 2.2. Preparation and Characterization of Oil Palm Ash Nanoparticles

The experimental design for the preparation of oil palm ash nanoparticles was adopted based on a previous study by [1] on the Taguchi L9 Orthogonal array method to obtain the optimum process parameters to produce oil palm bio-ash (OPB) nanoparticles with a horizontal ball milling technique. The most significant parameters for the milling operation of OPB nanoparticles are the size of balls, milling time, and milling speed.

The influence of the functional groups in the interaction between PF resin structure and OPA composition was investigated using the Fourier Transform Irradiation (FT-IR). The thermal stability of PF nanocomposites was evaluated using thermogravimetric (TGA). An X-ray Diffraction analyzer (XRD) was used to examine the crystalline structure in PF nanocomposites. Morphological analysis was performed to investigate the dispersion and distribution of OPB nanoparticles in PF resin nanocomposites.

The particle size analysis of the initial materials and milled powders was measured with a Malvern Mastersizer (Malvern Panalytical Ltd., Grovewood Road, United Kingdom) scirocco 2000 by dynamic light scattering measurements. In each measurement, the detector and laser were aligned, and the background was calibrated. Size distribution was quantified as the relative volume of particles in size bands presented as size distribution curves (Malvern Mastersizer Software v 5.60). Particle size distribution was described according to D-Values (D0.1, D0.5, and D0.9), corresponding to 10%, 50%, and 90% of the cumulative mass.

Transmission electron microscopy (TEM) was carried out with an EFTEM Libra (Carl Zeiss instrument, Oberkochen, Germany). The OPB nanoparticles were oven-dried before using the TEM to characterize the size and morphology of the particles. The OPB nanoparticles were prepared in acetone and dispersed with an ultrasonicator for 10 min. TEM analysis samples were obtained by placing a drop of colloidal dispersion containing OPB nanoparticles onto a carbon-coated copper grid. Embedded OPB nanoparticles were air-dried at room temperature before being examined under a TEM instrument under control conditions.

### 2.3. Preparation of PF Nanocomposite-Filled OPA Nanoparticles

In this study, the PF nanocomposites were prepared with 0%, 1%, 2%, 3%, 4%, and 5% of OPB nanoparticles (based on wt. % of phenol-formaldehyde resin). The size of OPB used in the nanocomposite preparation was 91 nm (average sizes). The OPA nanoparticle loading was limited to 5% because of void and agglomeration in the cured nanocomposites. These problems could lead to a reduction in the performance of PF nanocomposites [11]. Thus, the desirability of OPB loading in polymers should have limitations to produce a good nanocomposite. The OPB nanoparticle was dried at 105 °C for 24 h and cooled in the desiccator containing silica gel to prevent moisture absorption.

The preparation of neat PF resin involves pouring PF resin without OPB nanoparticles into the mould at room temperature. Afterwards, it was heated at 150 °C to polymerize the PF resin. Then, the PF resin was mixed with the desired amount (1%, 2%, 3%, 4%, and 5%) of OPB nanoparticles at room temperature for 30 min using a mechanical stirrer at 800 rpm. This approach afforded the flexibility in exercising independent control over reinforcement material and reinforcement geometry (size, morphology, and size distribution) [18]. Each mixture was prepared using five replicates to obtain enough samples for testing and analysis. The mixture was then degassed in a vacuum chamber for 5 min to remove bubbles. The mixture was then cast into a mould and left to cure in the oven at 150 °C for 30 min. An average of 6 h was used as the nanocomposite gel time, and once the nanocomposite was cured, it was removed from the mould and left in the conditioning room for 24 h. This type of resin only utilizes wood-based panels. The solid phase or in plate form of cured and hardened PF resin could not be obtained. Thus, this research focused more on the PF resin’s physical properties, enhancing crystallinity, thermal stability, and other properties. To evaluate the properties of nanocomposites developed in this study, physical, thermal, and morphology characterization were performed.

### 2.4. Characterisation of PF Nanocomposite

#### 2.4.1. Fourier Transform Infrared (FT-IR) Spectroscopy

Fourier transform infrared (FT-IR) spectroscopy was performed using a Shimadzu (Kyoto, Japan) FT-IR instrument with the KBr pellet technique. The PF-filled OPB nanoparticles nanocomposites and Potassium Bromide (KBr) were dried accordingly before disk preparation. In disk preparation using the mortar, the OPB nanoparticles were pounded with KBr until the homogenized mixture was formed. Then, the mixture was pressed until 500 MPa to obtain a disk form. The disk was carefully placed into the Shimadzu FT-IR instrument for FT-IR analysis. The FT-IR spectrum was set between 400 cm^−1^ and 4000 cm^−1^ for the analysis.

#### 2.4.2. Thermogravimetric Analysis (TGA)

TGA was carried out using Mettler Toledo (Columbus, OH, USA) Model TGA/DSC simultaneous analyzer with a heating rate of 20 °C/min from room temperature up to 900 °C under nitrogen atmosphere. Investigation of PF nanocomposites filled with OPB nanoparticles thermal stability was analyzed in powder form using approximately 5–7 mg/analysis.

#### 2.4.3. X-ray Diffraction Analysis (XRD)

Wide-angle X-ray diffraction (XRD) was carried out to identify changes in the PF resins’ crystalline structure with and without OPB nanoparticles. X-ray diffractograms were obtained using a Philips (Tokyo, Japan) PW1050 X-pert diffractometer, Cu-Ka1 radiation, at 40 kV, 25 Ma, and λ = 1.54 Å. The diffractograms were scanned from 2.5° to 90° (2θ) in steps of 0.02° using a scanning rate of 0.5 °C/min.

#### 2.4.4. Measurement of Internal Bonding Strength

**Figure 1:**
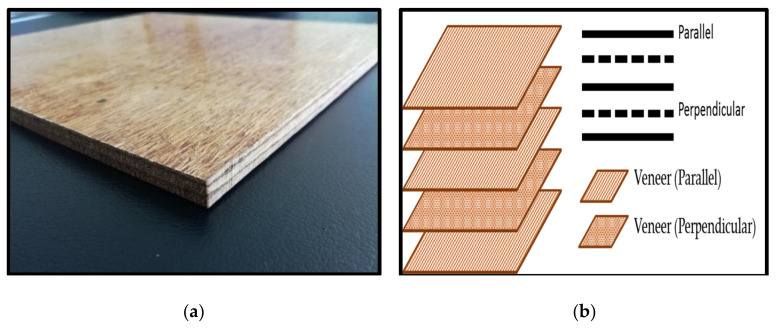
Schematic layering pattern of plywood composites. (**a**) Plywood composites and (**b**) The layering schematic.

The internal bonding strength of the PF nanocomposite was measured by using it as a binder in plywood. The plywood composites were prepared by using hand lay-up techniques for making a test sample. The schematic layering pattern of the 5-plywood veneer was shown in Figure 1 (a) Plywood composites and (b) The layering schematic. The layers were glued using phenol-formaldehyde resin-filled OPB nanoparticles with a glue spread rate of 400 g/m^2^ before being cold-pressed for 10 min. The cold press method is intended to develop consolidation between the surfaces of the raw materials. After that, they were then hot-pressed for 30 min. at a temperature of 150 °C, at approximately 200 bars (3000 psi) pressure. Finally, the plywood composites were left for cold pressing for 30 min before being cut into a standard dimension for internal bond strength.

The density profile of the samples along the thickness of panels was determined using Density Profiler Grecon model DA-X (Alfeld, Germany). The samples with a dimension of 50 × 50 × 10 mm were cut and maintained in a chamber at ambient temperature (25 ± 3 °C) and relative humidity of 30% (±2%) before testing. During scanning, the samples were inserted into the cassette holder for each batch scan and analyzed every 0.025 mm/s. In each case, the density profiles of the five samples were tested, and the average value tabulated.

IB strength was determined according to Japanese standards (JIS) using an Instron Testing Machine Model 5582 (Norwood, MA, USA). In this test, the length, width, and thicknesses of the samples were measured and recorded. The test sample was glued with hot melt adhesive to the two specimen holders, inserted into the testing equipment, and left for 24 h. A tension load of 2 mm/min was applied vertically to the sample face and the maximum load (*P*′) was measured at the time of failing force (breaking load of perpendicular tensile strength to the board). The sample was produced in triplicate, and standard deviation was used to measure the significant difference of the result.

#### 2.4.5. Field Emission Scanning Electron Microscopy (FESEM) and Energy-Dispersive X-ray Spectroscopy (EDX)

A FESEM-Carl Zeiss (Jena, Oberkochen, Germany) instrument was used to examine the morphological structure of the images of ground OPB and PF nanocomposite-filled OPB nanoparticles. A thin section of the sample was mounted onto an aluminium stub and sputter-coated with gold to enhance the conductivity of the test samples before the morphological examination. The FESEM micrographs were obtained under conventional secondary electron imaging conditions with an acceleration voltage of 5 kV. The EDX spectroscopy was recorded for all samples to investigate specific elements of phenolic nanocomposites.

## 3. Results and Discussion

### 3.1. Characterisation of Raw Oil Palm Bio-Ash (OPB) and Oil Palm Bio-Ash Nanoparticles

**Figure 2:**
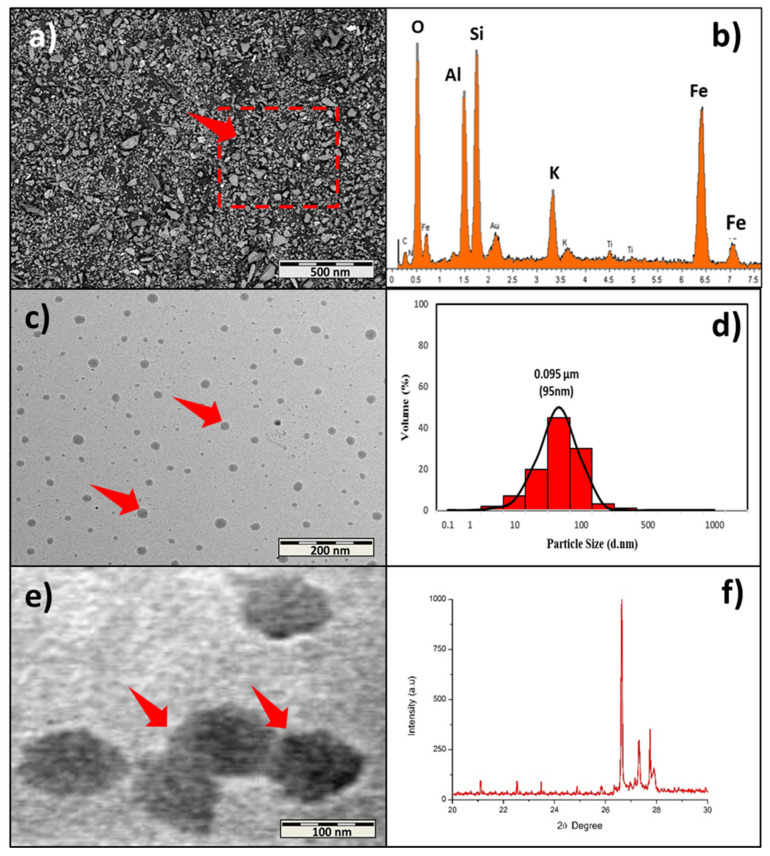
Properties of oil palm bio-ash nanoparticles. (**a**) FESEM morphology, (**b**) EDX element composition, (**c**) TEM micrograph of OPB distribution, (**d**) OPB nanoparticle distribution, (**e**) TEM micrograph of OPB, and (**f**) OPB XRD spectroscopy.

The morphology, particle distribution, and elemental composition of oil palm bio-ash (OPB) nanoparticles are shown in Figure 2. As seen from the TEM micrograph in Figure 2a, the OPB exhibited an irregular shape. In a previous morphology report by Abdul Khalil, Mahayuni [7], and Pa, Chik [31], it was revealed that OPB has irregular and angular shape structures. The characteristic shape of the OPB could provide a high surface area and ease of incorporation with polymers. In the EDX analysis illustrated in Figure 2b, Silicon (Si) was the main component in the OPB. Similar results of the high silicon (Si) component have been reported by Abdul Khalil et al. [32], indicating more than 25% of the overall OPB elements. Other elements, such as Ferum (Fe) and Aluminium (Al), showed an increment after the ball milling process. However, after the ball milling process, the silicon (Si) element still exhibited the highest element due to increased OPB nanoparticle surface area.

Results of OPB nanoparticles morphology and particle size distribution after the ball milling process are shown in Figure 2c,d. The micrograph indicates that the ball milling process with optimized conditions could produce OPB nanoparticles. The morphology of the OPB nanoparticles showed spherical particles with irregular shape structures. The distribution is shown in Figure 2d, demonstrating that the OPB nanoparticles had a mean distribution size of 91 nm (0.091 µm). A recent report by Tasnim, Du [33] also mentions the transformation of palm ash into smaller size, resulting in irregular and agglomerated palm ash shapes. Hence, these results indicate that the high surface area of the OPB and Silicon (Si) element could be an efficient reinforcement for phenol-formaldehyde (PF) nanocomposite properties.

X-ray Diffraction (XRD) testing is done to get diffraction patterns of the crystalline structures of the OPB nanoparticles. The X-ray diffraction (XRD) spectroscopy of OPB nanoparticles is shown in Figure 2f. At 26.7°, a high-intensity peak was observed, indicating the presence of silica, which was the main crystalline component in the OPB nanoparticles. Rizal et al. [34] and Mannan, Nagaratnam et al. [35] obtained silica dioxide as the main crystalline phase grounded palm ash. Simultaneously, the XRD spectrum presented two distinguishable sharp peaks between 27° and 28°, which probably indicated impurities or other metal oxides. Mostaghni and Abed [36] found that the state of impurities in Titanium Oxide (TiO_2_) was influenced by their concentration, band structure, and density. Besides, a previous study by Omar [37] also concluded that the presence of impurities would affect the peak intensity, 2*ϴ*position, and the shape of X-ray spectra. In addition, an observation by Alsubari, Shafigh [38] found that a minor crystalline phase occurred, which represented iron oxide (Fe_2_O_3_). However, it was suggested that impurities incapable of developing high peak intensity resulted in a crystalline structure due to the impurities attributed to foreign grains.

### 3.2. Characterisation of PF Nanocomposites-Filled Oil Palm Bio-Ash Nanoparticles

#### 3.2.1. Fourier Transform Infrared Spectroscopy (FT-IR)

**Figure 3:**
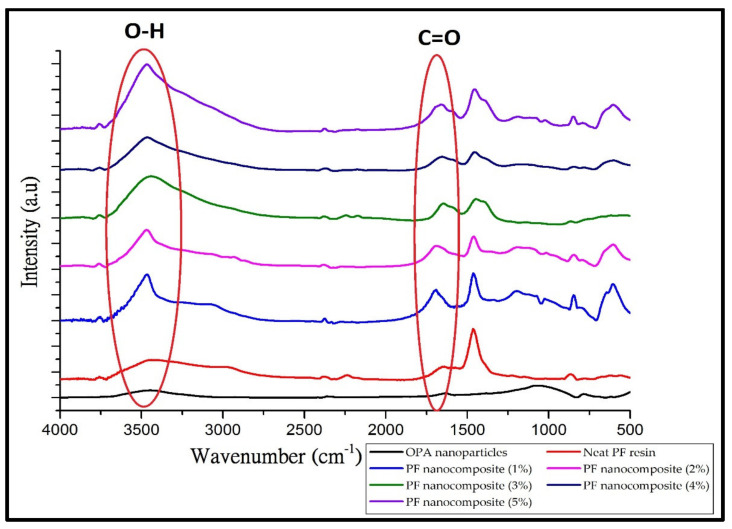
FT-IR spectra of neat PF resin, OPB nanoparticles, and PF nanocomposite-filled OPB nanoparticles (1% to 5%).

**Figure 4:**
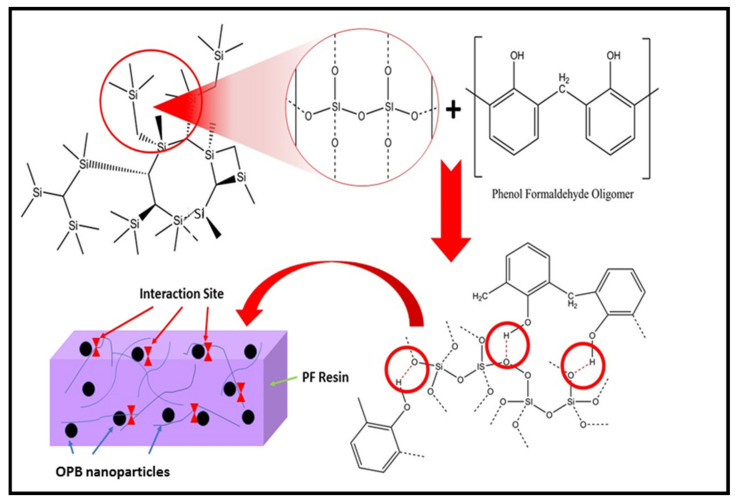
Hydrogen bonding mechanism of PF nanocomposite-filled OPB nanoparticles.

A comparison of the FT-IR spectrum of regular phenol-formaldehyde (PF) resin, OPB nanoparticle, and PF nanocomposite-filled OPB nanoparticles (1–5%) is shown in Figure 3. An FT-IR analyzer was used to measure the change of composition of the nanocomposite filled with OPB nanoparticles. The determination of molecular composition and structure depends on the absorbance level at each wavelength. The FT-IR spectrum, illustrated in Figure 4, provided useful information on intramolecular interactions in nanocomposites. There was a difference in the characteristics peaks of the FT-IR spectra, such as intensity value, peak broadening, and the presence of a new absorption peak. Figure 3 showed a change of the nanocomposite’s peak characteristic at band 3500 cm^−1,^ probably due to the reaction between OPB nanoparticles and phenol-formaldehyde at various times. The rise in peak intensity as the percentage of OPB nanoparticle increased indicating a growth of the Si-OH bond. The absorption bands observed at 3400 to 3500 cm^−1^ were stretching vibration of -OH and silanol hydroxyl groups of silica inside the OPB [7,39]. A hydroxyl functional group around 3500 cm^−1^ was reported by Ooi, Ismail [40], which is attributed to silanol (Si-OH) in the OPA particles.

These findings also agree with the observations by Paul, Satpathy [41] during the preparation of nanostructured materials from fly ash. Moreover, evidence also showed that reducing fly ash particle size increases the significant peak at 3448 cm^−1^. During the ball milling process to convert raw OPB to nano-size OPB, the breakdown of the silica structure leading to the formation of silanol resulted in the hydroxyl derivative of silane. The increase in the intensity may be due to the higher volume of –OH and a silanol hydroxyl group from OPB nanoparticles associated with phenolic hydroxyl bonds. The mechanism of hydrogen bonding between silica and phenol-formaldehyde resin in PF nanocomposite is shown in Figure 4.

The FT-IR spectra also revealed that once the OPB nanoparticles are incorporated with phenolic hydroxyl molecules, the bonding’s impact produces significant band broadening and stabilizes the peak intensity. The broadening peak and steady absorption frequency indicate asymmetric stretching and strength of the hydrogen bonding with the most energy and instability. In certain circumstances, the intermolecular hydrogen bonds between neighbouring molecules form a stable dimeric structure, a highly characteristic one, and lower mean absorption frequency [42]. The presence of a new absorption peak could be seen at band 1664 cm^−1^. The peak corresponds to the typical stretching of the C=O bond. For ordinary PF Resin, the peak at 1664 cm^−1^ shows lower stretching vibration C=O and the relative strength of the peak lower, compared with PF nanocomposite-filled OPB nanoparticles. The peak spawned for PF nanocomposite-filled OPB nanoparticles may probably be attributed to a reaction between elements of OPB nanoparticles such as silica and a phenol hydroxyl group from PF resin. Research by Chen Li et al. [43] and Vijayakumar Periadurai et al. [44] indicated that a reaction might exist between silica and resol compound nanocomposite, thus generating a steep peak in FT-IR spectra. Based on the obtained spectra, the broader bands of PF nanocomposite-filled OPB nanoparticles around 1500 cm^−1,^ and 3500 cm^−1^ were attributed to the stretching of –OH and bending vibration of O-H-O bonding. Usually, these bands were incorporated with water and water did not exist in OPB nanoparticles. This behaviour indicated a polymerization and transformation formation of –OH and O-H-O bonding in the nanocomposite [45].

#### 3.2.2. Thermogravimetric Analysis (TGA)

**Figure 5:**
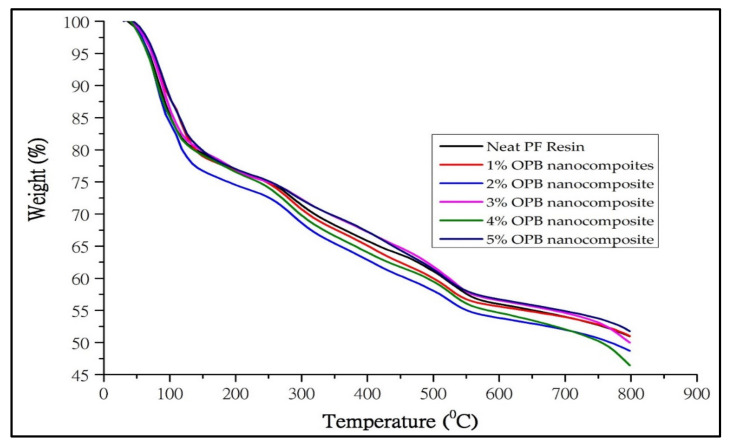
TGA thermograms of PF nanocomposite-filled OPB nanoparticles (0–5% OPA nanoparticles).

**Figure 6:**
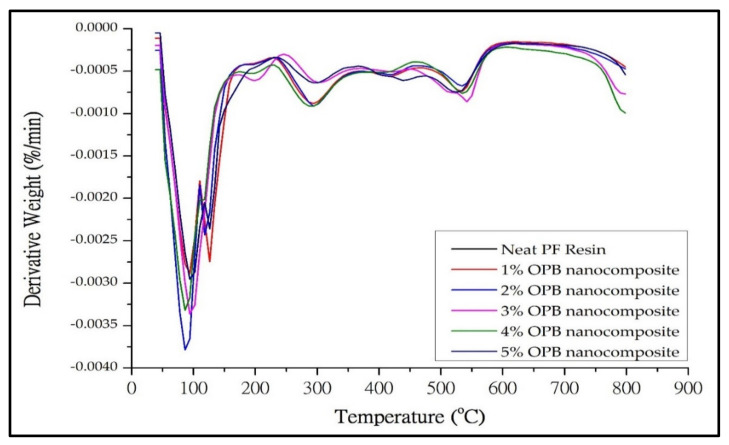
DTG thermogram of neat PF and PF nanocomposite-filled OPB nanoparticles with different loadings.

Thermogravimetric (TG) and derivatives thermogravimetric (DTG) of neat PF and PF nanocomposite are shown in Figure 5 and Figure 6, respectively. The addition of OPB nanoparticles into neat PF resin enhances the thermal stability behaviour of the PF nanocomposite, as shown in the initial degradation temperature (IDT), final degradation temperature (FDT), and residue content in the form of ash in Table 1. In general, nanocomposites’ weight loss was observed between 300–500 °C and terminated at 800 °C, where about 70% of the degradation occurs.

Particularly, the PF resin-filled OPB nanoparticles with different loadings showed a similar trend of thermolysis behaviour. This behaviour is attributed to the cross-linkage of the incorporated OPB nanoparticles with PF resin. In the first stage of weight loss, it was found that there was a small exothermic reaction in the range of 80–120 °C. This could be an evaporation process derived from water and formaldehyde that evolved due to increased temperature during the analysis [46]. PF resin usage as a nanocomposite matrix exhibited lower initial decomposition temperature changes, probably due to the composition properties of PF resin. This steady increase in initial degradation temperature of nanocomposites identified by Lele [47] and Alonso, Oliet, Dominguez, Rojo, and Rodriguez [29] was due to the loss of a volatile compound which contained low molecular weight materials, solvent, and some monomers. Research by Ooi et al. [48] also suggested that the hydroxyl groups in the chains of OPB nanoparticles tend to absorb moisture from the environment, resulting in degradation at elevated temperatures.

The results of the thermal analysis via TGA thermograms are expressed in Table 1. The initial thermal degradation of the nanocomposite shifts slightly towards the higher temperature range compared to pure PF composite. Compared to the ordinary PF composite, the initial degradation temperature of the nanocomposite rose to 21%. This evidence clearly shows that OPB nanoparticles enhance the thermal stability of the nanocomposite. In PF resin nanocomposites, the OPB nanoparticles have a reactive surface that provides a chemical connection into the PF resin matrix, resulting in a higher interaction network. This result is also consistent with Solyman et al. [49], who state that rubber nanoparticles’ reactive surfaces develop a higher crosslinked network. Simultaneously, the silica structure merged with PF resin was restricted to break at a lower temperature due to intermolecular hydrogen bonding. However, the restriction is over once the other compositions start to degrade at a higher temperature [30].

The second stage degradation of nanocomposites takes place at 290 °C up to 321 °C. From Table 1, the PF nanocomposite-filled OPB nanoparticles greatly show higher initial degradation temperature compared to ordinary PF resin. Nanocomposites filled with 1% OPB nanoparticles exhibited the highest initial degradation temperature (IDT). The higher IDT denoted by the nanocomposite was probably due to the homogenous distribution of OPB nanoparticles in the matrix, thus creating a better formation with PF resin components. The thermal stabilization could be developed due to the additional network created between OPB nanoparticles and PF polymer chains. Besides, the reinforcing effect of OPB nanoparticles could create complex polymer structures, develop crosslink density, and possibly produce conductive resistance.

Moreover, due to the effect of heat retardant of OPB nanoparticles, more energy is required during the degradation process, therefore enhancing the thermal stability of the nanocomposites [50,51]. Meanwhile, a higher percentage of OPB nanoparticles (2–5%) tend to agglomerate during the mixing process, resulting in a decrease in the surface interaction between the PF matrix and OPB nanoparticles. Consequently, OPB nanoparticles exhibited a lower effect on the heating barrier to protect the nanocomposite from decomposed, reducing thermal stability. Moreover, less energy will be needed to transform the substance polymer into the gas phase or char formation. According to Etemadi and Shojaei [52], increasing nanomaterial loading reduced the crosslinking density and created a non-uniform crosslink structure thermosetting system of nanoparticle-polymer interaction. The enhanced thermal stability, especially for IDT of nanocomposites, could be attributed to the introduction of OPB nanoparticles.

Meanwhile, the final degradation temperature (FDT) of nanocomposite-filled 1% OPB nanoparticles increased from 506–516 °C, possibly incorporating OPB nanoparticles with PF polymer chains. With the lower loading of OPB nanoparticles, there was a significant interaction between PF polymer chains and OPB nanoparticles, possibly impeding the oxidation of polymer chains and increasing the nanocomposites’ final degradation temperature. However, with the increase of OPB nanoparticle loading in nanocomposites, the final degradation temperature gradually decreased and became a comparable value after that. The decrease of FDT could be from the non-uniform dispersion of OPB nanoparticles throughout the PF matrix [53]. This behaviour will probably affect the thermal energy transfer process between OPB nanoparticles and PF polymer chains due to agglomeration build-up that decreased the low surface area of the OPB nanoparticles.

The last stage of the degradation process, which is preferably called the carbonization zone, occurs at 700 °C. Table 1 shows that the final ash content residue would increase a higher percentage of OPB nanoparticle loading. The experimental results were also similar to previous work by Zhu et al. [54]. The addition of a nanofiller into a neat polymer matrix would act as thermal insulation against the volatile compound during the degradation of the polymer matrix under thermal conditions. Even though the nanocomposites generate higher ash content at the final stage, the complexity of char formation itself could not affect the thermal stability since it was obtained at the end of the degradation process [55].

However, with a vast relative surface area of OPB nanoparticles, there was still great interaction between OPB nanoparticle components with PF molecular chains, which enhanced the thermal stability of nanocomposites compared to neat PF composite. Compared with the neat PF nanocomposite, degradation temperatures were observed to be higher. This phenomenon proved that any filler, either micron or nano size, would play a significant role in acting as thermal insulation to improve the poor thermal stability of the PF matrix. Based on Table 1, the final degradation temperature of PF nanocomposites could not increase progressively due to the OPB nanoparticles loading above a critical value. As reported by [28], who used copper nanoparticles as reinforcement in phenolic nanocomposites, the increase of copper nanoparticles content leads to stagnant degradation temperature due to the significant aggregation of copper nanoparticles. While in PF nanocomposites degradation, the final degradation temperature showed a slight reduction after the addition of OPB nanoparticles, and this behaviour was probably due to aggregation. Further aggregations certainly affected the structural surface of OPB nanoparticles and lowered the interaction bonding of the nanocomposite component. Hence, the PF nanocomposite could deteriorate and result in inferior performance with regards to thermal stability.

#### 3.2.3. X-ray Diffraction Analysis (XRD)

**Figure 7:**
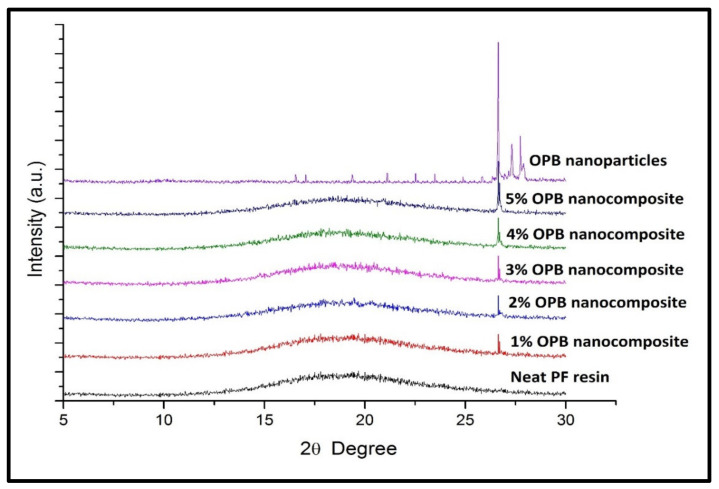
XRD spectroscopy of neat PF resin and PF nanocomposite-filled OPA nanoparticles.

The X-ray Diffraction (XRD) spectroscopy PF nanocomposite-filled OPB nanoparticles compared to neat PF resin are shown in Figure 7. To evaluate the degree of aggregation of OPB nanoparticles, also called crystallized structured in PF nanocomposites, the XRD profile of neat PF resin was used as a reference. According to XRD results, the neat PF resin displayed the absence of a peak in the XRD pattern and indicated that PF resin was an amorphous structure. Figure 7 shows the degree of crystallinity of PF nanocomposites, probably due to the presence of OPB nanoparticles. In addition, it was observed that there was a decrease in amorphous structure with the increase of OPB nanoparticle content. The change in the XRD pattern for PF nanocomposites samples showed that the intensity of the reflection peak increased progressively as the loading of OPB nanoparticles was introduced and mixed into PF resin. As the loading of OPB nanoparticles increased from 1 to 5%, the peak became sharper and more intense. In the 2θ range of 25 to 30°, the peak that appeared in the XRD spectrum of PF nanocomposite (1–5%) is mainly attributed to the presence of OPB nanoparticles. Similar results were obtained by Kaushik Singh [56] on modified montmorillonite (MMT) powder reinforced in the nanocomposite. The XRD analysis showed higher peak intensity (sharp) due to powder MMT’s highly crystalline behaviour. Furthermore, a diffraction line profile study by Scardi and Leoni [57] also suggested that the peak intensity of the XRD spectral indicated the degree of crystalline structure, homogeneity, and morphology of the material.

The broadening (width) of the XRD spectrum indicated the effect of lattice strain. In this case, OPB nanoparticle in PF nanocomposites, probably OPB nanoparticle, has formed a bigger crystal size, resulting in less of a broad peak and certainly developing a sharp peak, as presented in Figure 7. PF nanocomposite (1%) diffraction pattern showed a relatively lower peak intensity, indicating a low volume of OPB nanoparticles and low crystallinity. On the other hand, when the loading of OPB nanoparticles increased, the peak intensity of the XRD patterns simultaneously increased, indicating the growth of the crystal structure of OPB nanoparticles in PF nanocomposite. It could be assumed that an increase of crystallized components of OPB nanoparticles is expected to enhance the physical and mechanical properties of the nanocomposite.

#### 3.2.4. Measurement of Density Profile and Internal Bonding Strength

**Figure 8:**
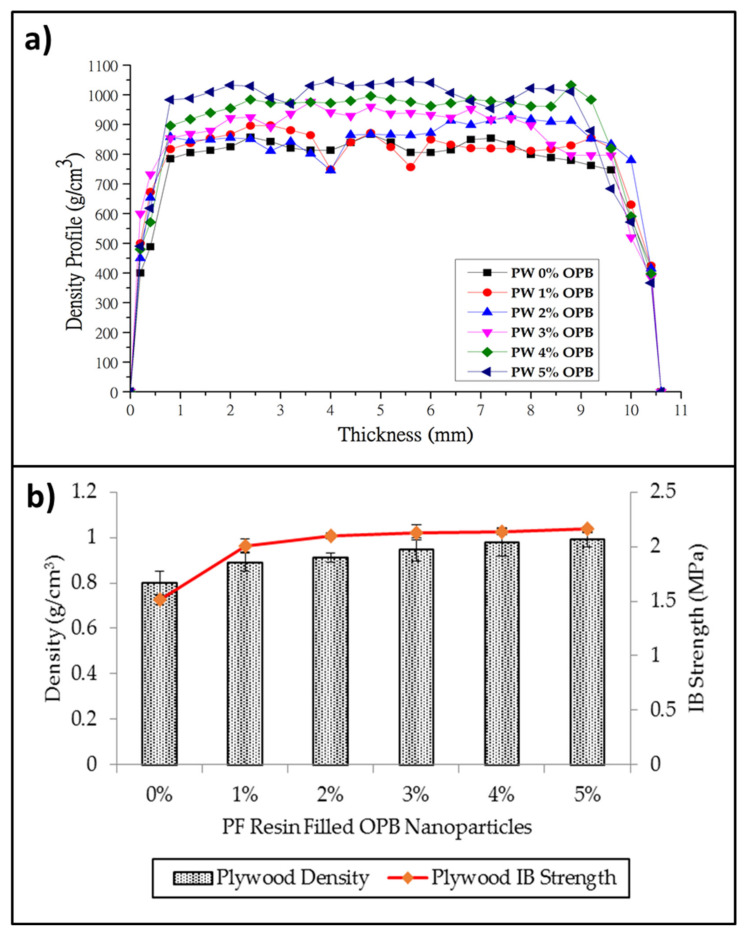
(**a**) Density profile of plywood with PF resin-filled OPB nanoparticles and (**b**) relationship between density and IB strength of plywood composites.

The density profile of plywood composites and the relationship of the average density of plywood composites with internal bonding strength in different loading of OPB nanoparticles are shown in Figure 8a,b, respectively. The PF resin as a binder in plywood composites has been used to prepare the plywood with different OPB nanoparticle loading (with 0%, 1%, 2%, 3%, 4%, and 5%). The plywood (PW) density profile with 10 mm ± 7 mm of average thickness is shown in Figure 8a. The PW with 5% of OPB showed the highest and most uniform density than other PWV with lower OPB content. From the density profile, it could be seen that higher OPB content in PW resulted in uniform density distribution. The uniformity of PW with higher OPB content was due to veneer bonding. This could best be explained by the fact that PF resin with higher OPB content could permeate into gaps and porous space between veneers, and then be cured to form an interlock between veneer layers. Therefore, the density profile of PW with higher OPB content showed a lower frequency of peak altitude and enhanced density distribution across the PW composites.

The relationship between density and internal bond (IB) of the plywood composite was displayed in Figure 8b. From the results, the IB of the plywood composite without OPB nanoparticles was low at the density of 0.8 g/cm^3^ compared to IB of plywood composites applied with OPB nanoparticles. In general, the IB of plywood composites tends to increase with increasing plywood composite density. From the results, it can also be seen that the application of PF-filled OPB nanoparticles on the veneer did not only improve the bonding quality but also slightly increase the density of the plywood composites. PF resin-filled OPB nanoparticle loading could be attributed to the interpenetrated network, which caused the OPB nanoparticles to fill in the plywood porosity veneer during the curing process.

Meanwhile, the higher content of OPB nanoparticles in PF resins affects the interface bonding between PF resins and layering panels. The possible reason for the enhanced IB of the plywood composites was that reinforcement with OPB nanoparticles improved the crosslink density of the PF resin, which helped the PF resin to be cured throughout the panel [58]. The difference in the self-bonding ability between PF resin-filled OPB nanoparticles and veneer might be caused by the interaction and chemical structure components’ difference in IB performance. The high degree improvement was also found by [59]—the nanoparticle compounds can deeply penetrate the wood and effectively alter its surface chemistry, resulting in better performance in physical and mechanical strength. Thus, this result suggested that OPB nanoparticles would play an effective role as an enhancer in plywood composites properties.

#### 3.2.5. Field Emission Scanning Electron Microscope (FESEM) Analysis

**Figure 9:**
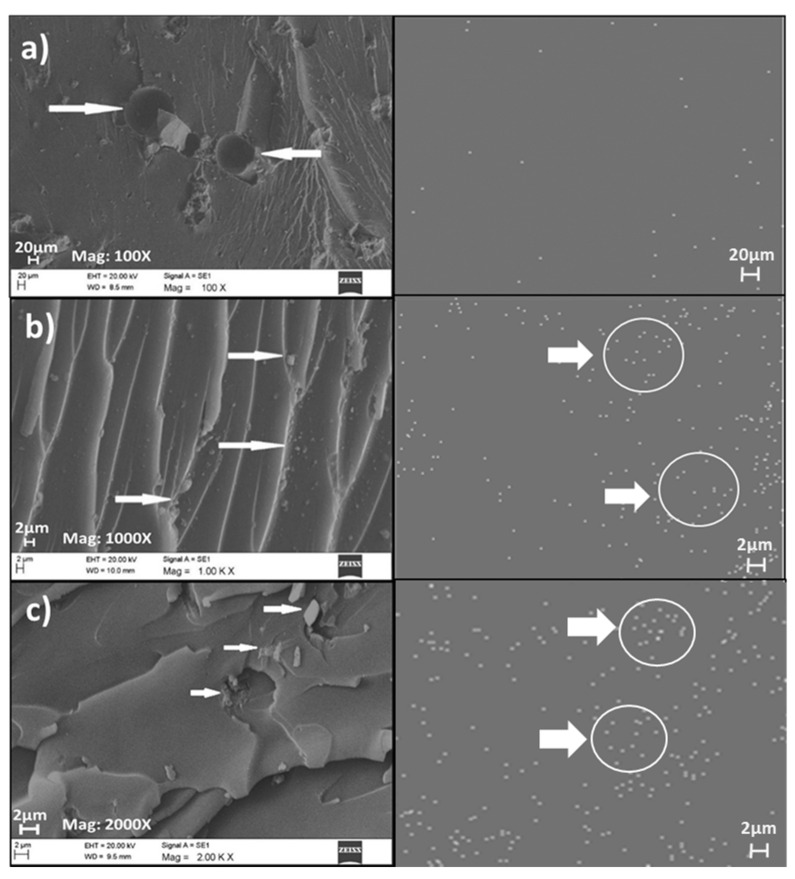
FESEM micrograph and elemental distribution mapping of (**a**) neat PF resin, (**b**) PF nanocomposite-filled 1% OPB nanoparticles, and (**c**) PF nanocomposite-filled 2% OPB nanoparticles.

**Figure 10:**
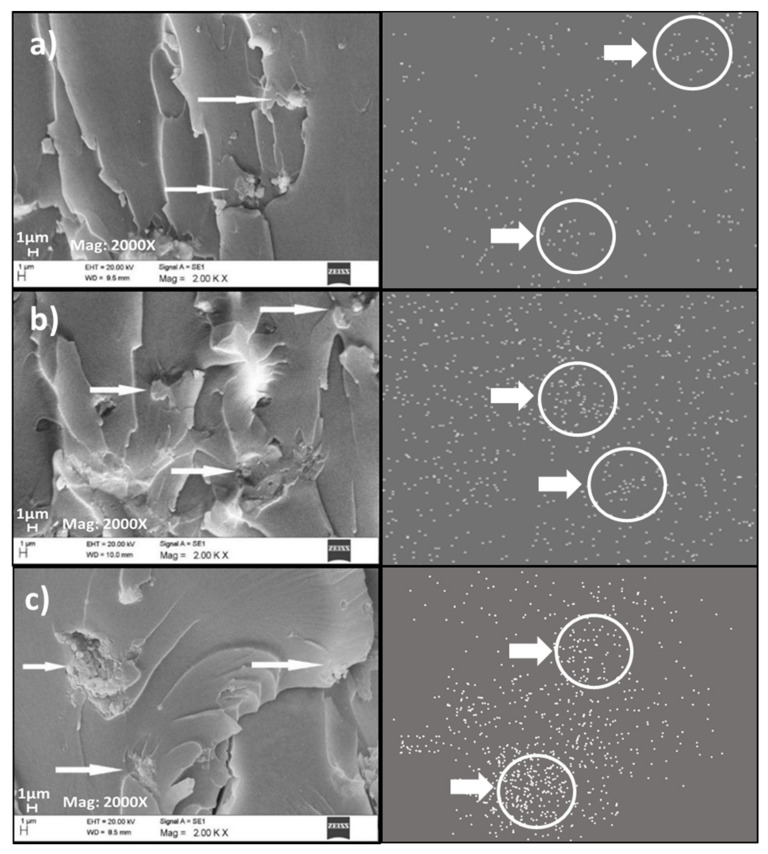
FESEM micrograph and elemental distribution mapping of (**a**) PF nanocomposite-filled 3% OPB nanoparticles, (**b**) PF nanocomposite-filled 4% OPB nanoparticles, and (**c**) PF nanocomposite-filled 5% OPB nanoparticles.

The morphological properties of neat PF and PF nanocomposite-filled FESEM investigated OPB nanoparticles are shown in Figure 9 and Figure 10. The FESEM micrograph and elemental distribution mapping were performed to understand the distribution of OPA nanoparticles in PF resin matric and gain an idea about the degree of agglomeration and aggregation and distribution behaviour of OPB nanoparticles. The FESEM micrograph of neat PF and PF nanocomposite-filled 1%, 2%, 3%, 4%, and 5% OPB nanoparticles were performed. FESEM micrograph of neat PF resin, PF nanocomposite with 1% and 2% OPB nanoparticles loading are presented in Figure 9a–c.

Figure 9a–c illustrates the FESEM of the neat PF resin matrix and PF nanocomposite-filled 1 and 2% OPB nanoparticles after curing for 1 h at 150 °C. Figure 9a shows that the surface was clear, and impurities were embedded in the neat PF resin. Furthermore, it shows evidence of void and porosities, probably due to water vapour that got trapped during heat curing. The process of void and water vapour bubbles development originated from the condensation cure of the phenolic resin. Besides, polymerization of phenolic resin with heat released the water molecules and formed macro and microvoids in the cured PF resin [60,61]. This behaviour shows that neat PF resin exhibits typical brittleness due to the incorporated OPB nanoparticles not appearing [62]. FESEM images of PF resin nanocomposite-filled 1%, and 2% OPB nanoparticles are shown in Figure 9b,c respectively. The micrograph showed the presence of OPB nanoparticles in the PF resin matrix. It can be seen that the OPB nanoparticles are partially distributed in PF resin with 1% OPB nanoparticle loading.

Meanwhile, with 2% OPA loading, the behaviour of the OPB nanoparticles tends to agglomerate due to an increase of surface energy between themselves. The homogenous dispersion of OPB nanoparticles in PF nanocomposites without an obvious aggregate phase separation could be seen in Figure 10b compared to Figure 10c. Figure 10a–c illustrated the FESEM images of the PF nanocomposite filled with 3%, 4%, and 5% OPB nanoparticles loading, respectively.

Figure 10a–c indicated that OPB nanoparticles dispersed less homogenously than PF nanocomposite-filled 1% and 2% OPB nanoparticle loading in Figure 10b,c. It was assumed that increases in OPB nanoparticle loading progressively escalated the surface energy of OPB nanoparticles and influenced their agglomeration. Furthermore, the agglomeration of OPB nanoparticles will worsen PF nanocomposite performance, particularly with regard to mechanical properties. Besides, agglomeration occurs because an excess of OPB nanoparticles could create a stress concentration zone that might act as a crack initiator. Kιzιlcan and Özkaraman [61] utilized clay as nano-reinforcement in a clay nanocomposite. The agglomerate of clay could be reduced by using high shear mixing and ultra-sonication. However, proper technique needs to be used to avoid an increase in bubbles and space due to excess mixing rates.

Based on Figure 10b,c the FESEM image displayed critical agglomeration of OPB nanoparticles, probably due to increased PF viscosity and poor distribution of OPB nanoparticles. A previous study by Lin, Fang, Li, Xi, Zhang, and Sun [28] reported that the matrix’s viscosity could be a major factor in nanoparticle dispersion. Significant dispersion of nanoparticles can be achieved when the pre-polymer is of low viscosity, which helps the nanoparticles easily and evenly disperses into PF resin. The agglomeration and aggregation of OPB nanoparticles in PF resin are inevitable due to their high specific surface and high surface energy [52]. Therefore, proper OPB loading and mixing techniques must improve the homogenous dispersion of OPB nanoparticles in the PF resin matrix.

The improved performance of PF nanocomposites has potential use in the wood-based industry. This work opened a gate to utilise agriculture waste transformation into nanomaterials incorporated with adhesives or resin to produce low-cost and environmentally-friendly products. Different products can be generated using different nanoparticle sizes. A further study on the effect of nanoparticle size on the properties of PF nanocomposite can be conducted.

## 4. Conclusions

The preparation and incorporation of different percentage loading of OPB nanoparticle as a filler in resin was successfully conducted without miscibility enhancement. The morphology of the nanocomposite confirmed the dispersion of nanoparticles in the PF resin nanocomposite. The properties of the resulting nanocomposites show improved internal bonding strength with nanoparticle addition, compared with the neat resin. In addition, the thermal stability of the nanocomposite was observed to increase due to the incorporation of nanoparticles. The enhancement in its properties was probably due to forming a bond between the matrix and the filler, as shown by the FT-IT studies. Furthermore, the crystallinity of the nanoparticles is shown in the Xrd result. The PF nanocomposites’ improved performance in this study could lead to improved wood-based industry and multifunctional products for various applications. The use of agricultural waste in functional nanomaterials could reduce the pollution problem and the cost of production of environmentally friendly products.

## Figures and Tables

**Table 1:** Thermal properties of nanocomposite-filled OPA nanoparticle.

Nanocomposite-Filled OPA Nanoparticles	Initial Degradation Temperature (IDT)	Final Degradation Temperature (FDT)	The Residue (%)
Neat PF Resin	265.06	396.14	42.79
1% OPB Nanocomposite	321.32	516.60	51.49
2% OPB Nanocomposite	301.91	495.61	51.78
3% OPB Nanocomposite	299.69	483.05	52.41
4% OPB Nanocomposite	302.57	503.41	52.11
5% OPB Nanocomposite	302.66	474.31	53.02

## Data Availability

Not applicable.
